# A long non-coding RNA HOTTIP expression is associated with disease progression and predicts outcome in small cell lung cancer patients

**DOI:** 10.1186/s12943-017-0729-1

**Published:** 2017-10-17

**Authors:** Yanqin Sun, Yuanyuan Zhou, Yifeng Bai, Qiongyao Wang, Jiarong Bao, Yingshan Luo, Ying Guo, Linlang Guo

**Affiliations:** 1 Department of Pathology, Zhujiang Hospital, Southern Medical University, Guangzhou, 510282 China; 2 Department of Pathology, Guangdong Medical University, Dongguan, China; 3 Department of Pathology, Shunde Hospital, Southern Medical University, Guangdong, China; 4 Department of Oncology, Sichuan Academy of Medical Sciences and Sichuan Provincial People’s Hospital, Chengdu, China; 5 Department of Organ transplantation, Zhujiang Hospital, Southern Medical University, Guangzhou, 510282 China

**Keywords:** Small cell lung cancer (SCLC), Hottip, Proliferation

## Abstract

**Background:**

Despite progress in treatment of small cell lung cancer (SCLC), the biology of the tumor still remains poorly understood. Recently, we globally investigated the contributions of lncRNA in SCLC with a special focus on sponge regulatory network. Here we report lncRNA HOTTIP, which is specifically amplified in SCLC, is associated with SCLC proliferation and poor prognosis of patients.

**Methods:**

RT-qPCR was used to investigate the expression of HOTTIP in SCLC tissues and cell lines. The role of HOTTIP in SCLC cell proliferation was demonstrated by CCK8 assay, colony formation assay, flow cytometry analysis and in vivo SCLC xenograft model in nude mice through HOTTIP loss- and gain-of-function effects. Western blot assay was used to evaluate gene expression in cell lines at protein level. RNA pull-down, Mass spectrometry and RNA binding protein immunoprecipitation (RIP) were performed to confirm the molecular mechanism of HOTTIP involved in SCLC progression.

**Results:**

We found that HOTTIP was overexpressed in SCLC tissues, and its expression was correlated with the clinical stage and the shorter survival time of SCLC patients. Moreover, HOTTIP knockdown could impair cell proliferation, affect the cell cycle and inhibit tumor growth of mice, while HOTTIP overexpression might enhance cell proliferation and cell cycle in vitro and in vivo. Mechanistic investigations showed that HOTTIP functions as an oncogene in SCLC progression by sponging miR-574-5p and affecting the expression of polycomb group protein EZH1.

**Conclusions:**

Overall, we identified that HOTTIP was involved in SCLC tumorigenesis through the ceRNA network “HOTTIP/miR-574-5p/EZH1”. Our findings not only illuminate how HOTTIP confers an oncogenic function in SCLC pathogenesis, but also underscore a novel gene expression governing hallmarks in the disease.

**Electronic supplementary material:**

The online version of this article (10.1186/s12943-017-0729-1) contains supplementary material, which is available to authorized users.

## Background

Small cell lung cancer (SCLC), which accounts for approximately 15% of lung cancer, is one of the most malignant diseases world-wide, with a high mortality [1]. Unlike most malignancies, the majority of patients suffering from SCLC are diagnosed at advanced stages accompany with early and distant metastasis [2]. Hence, an intensive research of molecular mechanism involved in SCLC pathogenesis is vital for the identification of diagnostic and therapeutic targets.

Non-coding RNAs account for more than 90% of the transcriptome without protein-coding potential. Therein, microRNAs (miRNAs, 19–25 nucleotides) have been extensively studied, thousands of which regulate up to 30% of their protein-encoding target genes [3]. Additionally, long non-coding RNAs (lncRNAs) with length over 200 nucleotides, have been identified to play crucial regulatory roles in tissue differentiation, proliferation, migration, invasion and apoptosis [4]. Recent studies have indicated that, lncRNA could regulate the expression of some key oncogenes or tumor suppressor genes and affect the occurrence and development of tumor through lncRNA-miRNA or lncRNA-mRNA interaction. Competing endogenous RNA (ceRNA) was first proposed by Pandolfi PP et al. and had been proven as one of the important mechanisms in lncRNA regulation [5, 6]. Therefore, several lncRNAs including HOTAIR, HOXA11-AS, MALAT1 and H19 have been confirmed as ceRNAs in tumorigenesis, lung cancer is included [7]. Despite identification of these lncRNAs, the prevalence and functional significance of lncRNA-mediated sponge regulation and their relevant targets in SCLC remain unclear.

The lncRNA HOTTIP (HOXA transcript at the distal tip), a newly identified lncRNA, located at the 5′ end of the HOXA cluster, which is a key locus control element of HOXA genes and distal identity, and is brought into close proximity to the 5′ HOXA genes by chromosomal looping [8]. Existing studies have found that, large domains of HOX gene cluster are occupied by Polycomb Repressive Complex 2 (PRC2), which maintains the repressive histone marker H3K27me3 [9]. PRC2 is a sort of important gene expression regulatory elements, which mediates H3K27 methylation regulation and cause polycomb gene (PcG) silencing, then close down some tumor suppressor genes and cause tumorigenesis [10]. PRC2 is composed of several members including SUZ12, EZH1, EZH2 and EED. Among them, enhancer of zeste homolog 1 (EZH1) protein is an key methyltransfer enzyme in PRC2 components, which have RNA binding domains and may be combined with HOTAIR as well as other lncRNAs [11]. RIP-sequencing identified thousands of lncRNAs including HOTAIR, whose interaction with PRC2 are essential for their recruitment [12]. HOTTIP is also found to be associated with PRC2 and play a critical role in various malignancies including hepatocellular carcinoma, pancreatic cancer, gastric cancer, colorectal cancer and so on [13].

Until now, the prevalence and functional significance of lncRNA-mediated sponge regulation and their relevant targets in SCLC are unclear. Our previous study based on lncRNA array has shown that HOTTIP is up-regulated in SCLC multidrug resistant cells (H69AR) compared to its parental H69 cell (data not shown). We further found that HOTTIP was not only related to SCLC chemo-resistance, but also closely associated with SCLC cell proliferation. In the present study, we attempt to investigate HOTTIP-mediated sponge regulatory network of protein-coding driver genes in SCLC pathogenesis. We also validate the tumor-promoting function of HOTTIP predicted to serve as miRNA sponge and positively regulate the expression of EZH1. Our study suggests an important role of HOTTIP in SCLC development and implied a therapeutical strategy of manipulating oncogene function through modulating HOTTIP-mediated sponge regulation.

## Results

### HOTTIP is up-regulated in SCLC and associated with poor prognosis of SCLC patients

**Fig. 1:**
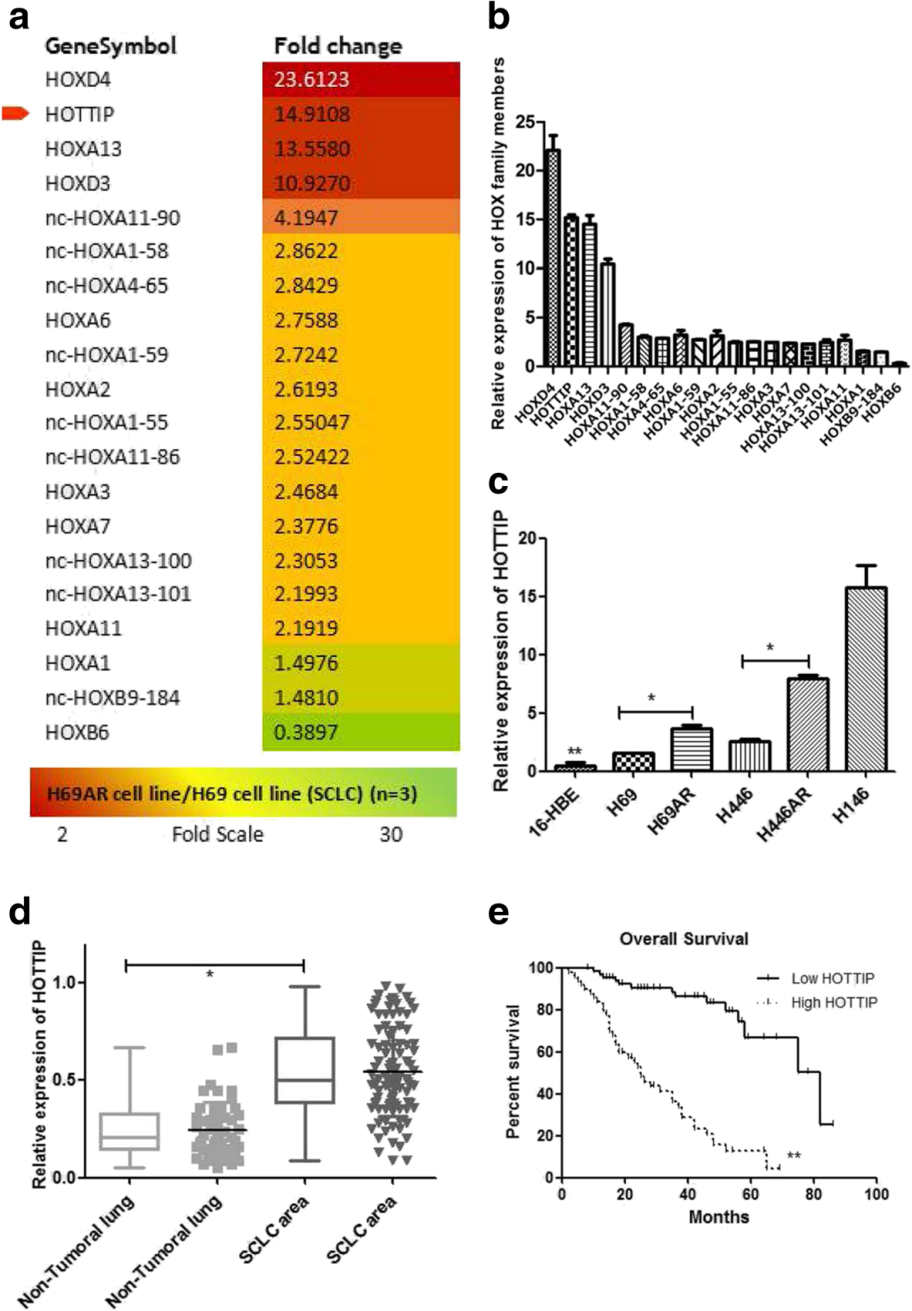
Gene differential expression array analysis and confirmation in SCLC cell lines and FFPE tissues. **a** & **b** Differentially expressed HOX genes were involved. **c** The level of HOTTIP expression in cell lines showed differential expression in 5 SCLC cell lines and the normal human bronchial epithelial cell line (16-HBE). **d** The level of HOTTIP expression in SCLC clinical tissues and the non-tumoral lung tissues. **e** Patients with high HOTTIP expression had a significantly poorer prognosis than those with low HOTTIP expression.*, *P* < 0.05; **, *P* < 0.01

Gene expression array analysis on H69 and H69AR cell lines showed 1443 differentially expressed lncRNAs statistically significant, their functions are involved in cell cycle gene regulation, apoptosis, enzyme activity regulation, metabolism, signal transduction activity and so on (Additional file 1: Fig. S1A–B). Among them, 20 HOX family members including lncRNA HOTTIP were up-regulated more than 10-fold changes in H69AR compared to H69 cell (Fig. 1a-b), which were validated in several SCLC cell lines by RT-qPCR (Fig. 1c). To further confirm these results, the level of HOTTIP expression was determined in SCLC clinical samples by RT-qPCR. Among them, 50 biopsy SCLC tissues and the control non-tumoral lung tissues were collected from patients before chemotherapy at Shunde Hospital of Southern Medical University. HOTTIP Expression was significantly up-regulated in SCLC tissues compared with non-tumoral lung tissues (Fig. 1d). Examination of the correlation between HOTTIP expression and clinical pathological features showed that HOTTIP was correlated with disease stage and median survival (Table 1). With regard to overall survival, patients with high HOTTIP expression had a significantly poorer prognosis than those with low HOTTIP expression (Fig. 1e). These results above imply a potential role of HOTTIP as a novel bio-marker for SCLC progression.

**Table 1:** Clinicopathological data of the SCLC studied cohort

Patients Characteristics	HOTTIP expression			
	**–**	**+**	χ^2^	*P* Value*
All cases (*N = 115*)	27	88		
Age			0.5957	0.4402
≤ 56	10	40		
> 56	17	48		
Gender			0.4183	0.5178
Male	15	55		
Female	12	33		
Disease stage			9.6084	0.0019
Limited disease (LD)	20	33		
Extensive-stage disease (ED)	7	50		
Median Survival (5-36 months)			23.5898	<0.001
Survival	19	18		
Death	8	70		

*For analysis of correlation between of HOTTIP levels and clinical features, Fisher’s Exact Test were used. Results were considered statistically significant at *P < 0.05*

### Manipulation of HOTTIP levels in SCLC cell lines

**Fig. 2:**
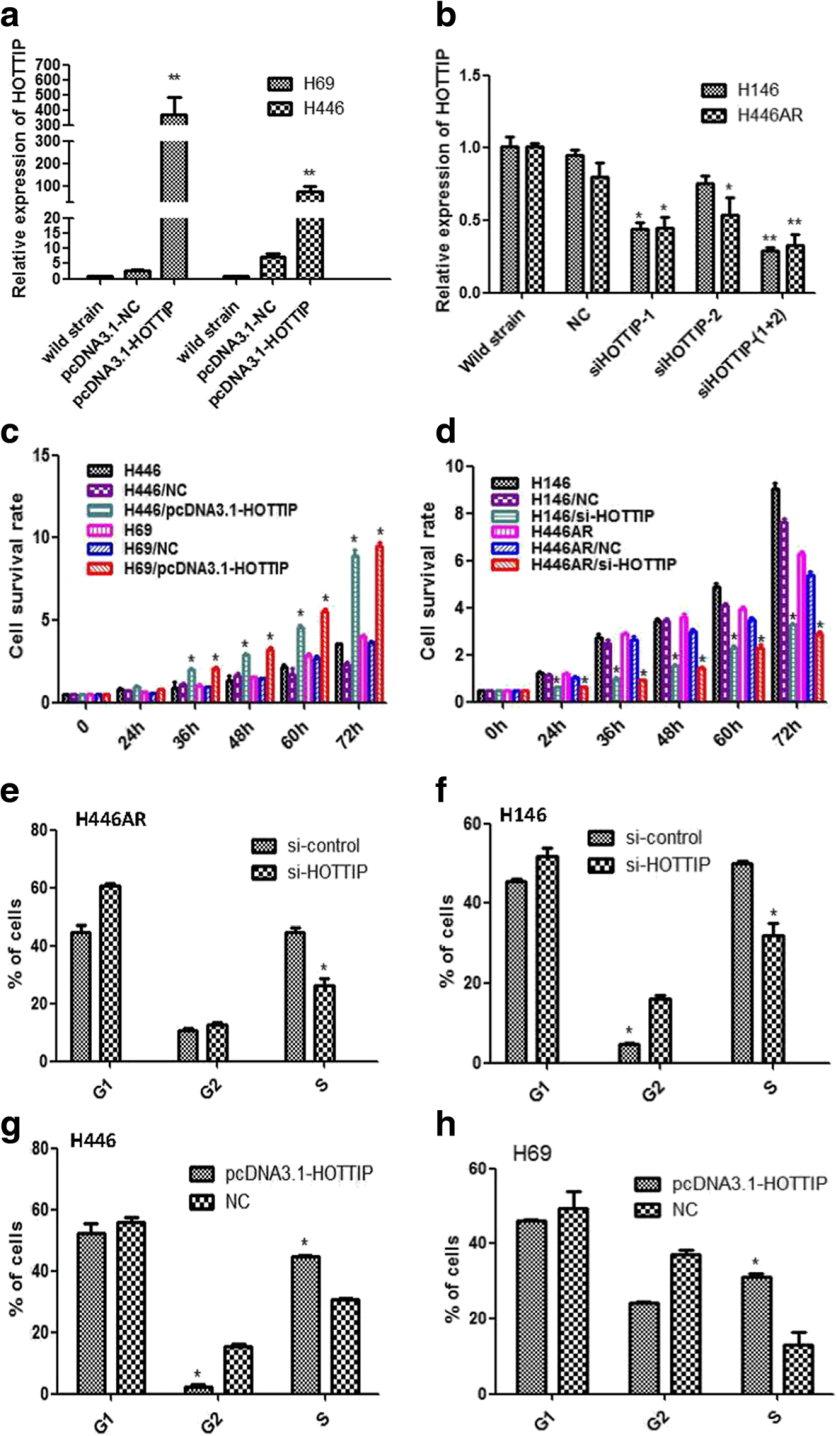
HOTTIP may regulate SCLC cell proliferation and cell cycle. **a** A pcDNA3.1-HOTTIP expression vector was transfected successfully into H69 and H446 cells, respectively. **b** HOTTIP RNAi sequences were transfected into H146 and H446AR cells, respectively. **c** CCK8 assay was used to detect the effect of HOTTIP on SCLC cell proliferation after HOTTIP overexpression. **d** CCK8 assay was used to detect the effect of HOTTIP on SCLC cell proliferation after HOTTIP knockdown. **e** & **f** Flow-cytometric analysis was used for cell cycle detection after HOTTIP knockdown in H446AR and H146 cells. **g** & **h** Flow-cytometric analysis was used for cell cycle detection after HOTTIP overexpression in H446 and H69 cells. *, *P* < 0.05; **, *P* < 0.01

HOTTIP expression was examined in SCLC cell lines (H69, H446, H146, H446AR, H69AR) and the normal human bronchial epithelial cell lines (16-HBE) by RT-qPCR. Among them, HOTTIP expression in H146 and H446AR are significantly higher than those in H69 and H446, respectively. Similarly, HOTTIP expression in either of the four SCLC cell lines is higher over 16-HBE cell (Fig. 1c). Hence, we used H146 and H446AR cells for loss-of-function experiments, while H69 and H446 cells were used for gain-of-function experiments. A pcDNA3.1-HOTTIP expression vector, HOTTIP siRNA sequences as well as the negative controls were transfected into H69/H446 and H146/H446AR cells, respectively, the effects of overexpression or interference were validated by RT-qPCR at 24 h after transfection (Fig. 2a-b).

### HOTTIP regulates SCLC cell proliferation in vitro as well as the cell cycle

To comprehensively investigate the role of HOTTIP in SCLC progression, we additionally established stable HOTTIP knockdown H146 and H446AR cell lines by lentivirus infection (Additional file 1: Fig. S1C-D). CCK-8 assays revealed that HOTTIP overexpression in H69/H446 cells resulted in much increased cell proliferation and a high cell survival rate (Fig. 2c). However, knockdown of HOTTIP reduced cell proliferation compared with negative controls in both cell lines (Fig. 2d). To further investigate the growth inhibition observed following HOTTIP knockdown, cell-cycle profiles of HOTTIP knockdown cells and negative controls were carried out using flow cytometry. Suppression of HOTTIP led to a decrease in the number of cells in the S-phase and an unstable cell number in the G2-phase (Fig. 2e-f, Additional file 2: Fig. S2D), while overexpression of HOTTIP resulted in an increase in the S-phase and a decrease in the G2-phase of cell number (Fig. 2g-h, Additional file 2: Fig. S2E).

**Fig. 3:**
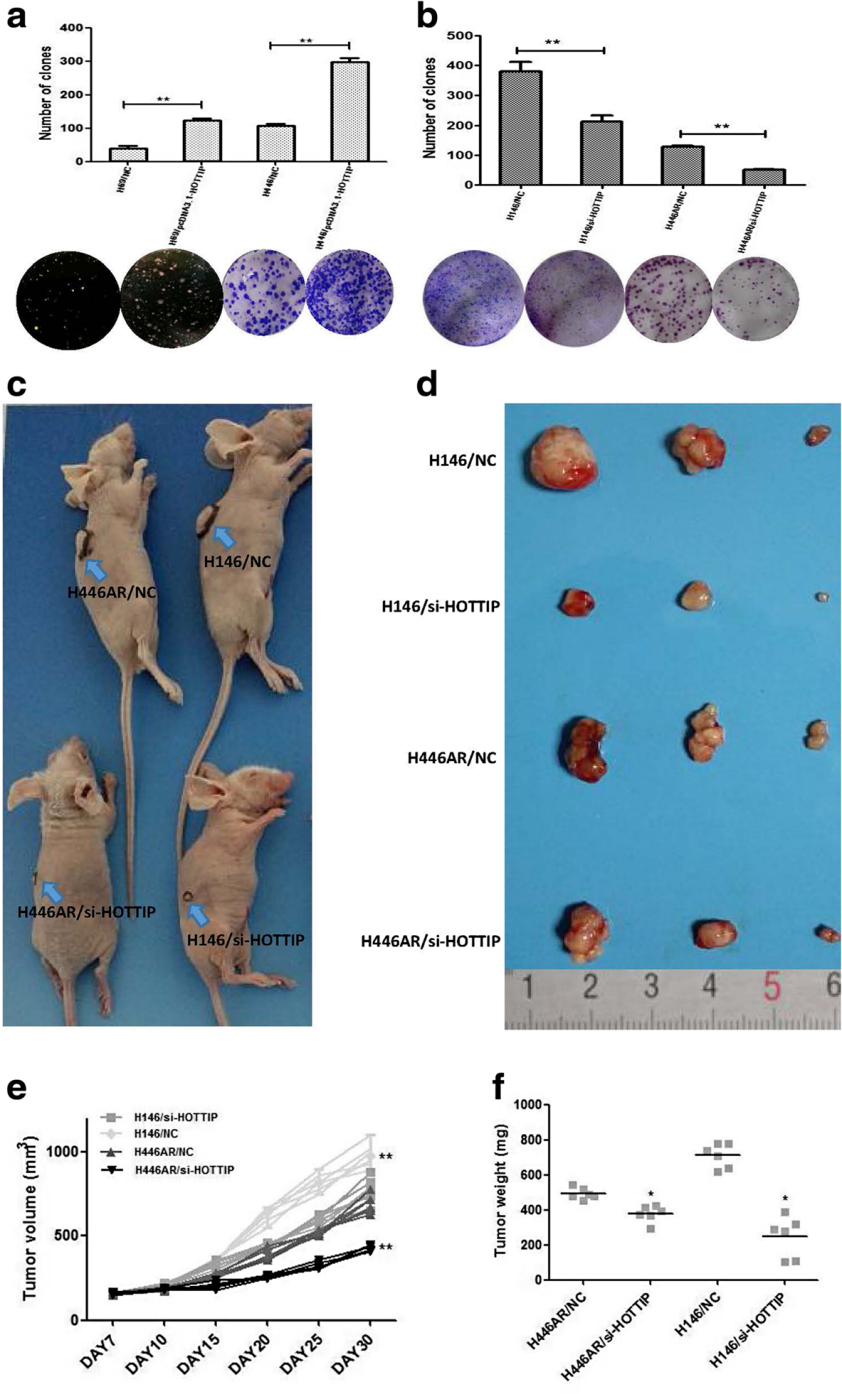
HOTTIP may regulate SCLC cell proliferation in vitro and xerograft tumor growth of in vivo. **a** Plate and soft agar colony formation assay was used to detect the effect of HOTTIP overexpression on SCLC cell proliferation in vitro. **b** Plate colony formation assay were used to detect the effect of HOTTIP knockdown on SCLC cell proliferation in vitro. **c** Images of nude mice with xerograft tumor in each group. **d** Excised tumors’ image from nude mice in each group. **e** Volume change curve of each group measured on the indicated days. **f** Tumor weights of each group were determined. *, *P* < 0.05; **, *P* < 0.01

Furthermore, plate colony forming assay and soft agar colony forming assay (for suspension-cultured H69 cell) were used to investigated the effect of HOTTIP on cell proliferation, HOTTIP overexpression increased the colony formation ability of H446/H69 cells (Fig. 3a), while HOTTIP knockdown inhibited the colony formation ability of H446AR/H146 cells (Fig. 3b).

### HOTTIP regulates SCLC cell growth in vivo

The effect of HOTTIP to confer SCLC biology was further examined using an in vivo SCLC xenograft model in nude mice. As shown in Fig. 3c and d, tumor growth was most significantly inhibited in mice following HOTTIP knockdown treatment either in H146 cell or in H446AR cell compared with the NC groups. After subcutaneous injection for 15 days, the mean tumor volume for the NC groups were more and more markedly larger than their HOTTIP knockdown treating groups, respectively (Fig. 3e). As expected, the tumor weight statistic of excised tumor showed a similar trend to that of tumor volume (Fig. 3f).

### HOTTIP may be involved in SCLC biology by a miRNA-lncRNA-mRNA regulatory network

There were 81 miRNAs related closely to SCLC biology by miRNA microarray, among them 37 miRNAs expressed higher in H69 cell compared to H69AR cell, while 44 miRNAs expressed lower in H69 cell compared to H69AR cell (Additional file 1: Fig. S1E-F, Table 2). Using the bioinformatics website RNA22-seq (https://cm.jefferson.edu/), 5 miRNAs including miR-574-5p were predicted that have targeted binding relationship with HOTTIP and have been testified by RT-qPCR (Additional file 1: Fig. S1G).

**Table 2:** Differentially expressed miRNAs in H69/H69AR cells

No.	Probe_ID	H69-cy3	H69AR-cy3	log2 (H69AR/H69)
		Signal	Signal	
1	hsa-miR-375	6454.34	20.05	−8.31
2	hsa-miR-200b	6273.58	23.22	−8.19
3	hsa-miR-335*	3835.09	13.18	−8.07
4	hsa-miR-335	3663.11	13.79	−8.04
5	hsa-miR-224	4.35	866.73	7.70
6	hsa-miR-143	4.36	789.54	7.41
7	hsa-miR-216b	1367.48	7.65	−7.41
8	hsa-miR-145	6.14	890.69	7.06
9	hsa-miR-100	43.57	4656.66	6.64
10	hsa-miR-200c	17,307.45	278.54	−5.94
11	hsa-miR-217	448.40	6.73	−5.89
12	hsa-miR-493*	468.53	9.02	−5.75
13	hsa-miR-7	8780.53	174.39	−5.65
14	hsa-miR-432	2116.51	42.31	−5.65
15	hsa-miR-199a-3p	163.81	7822.89	5.61
16	hsa-miR-216a	305.13	7.61	−5.33
17	hsa-miR-487b	593.66	15.51	−5.26
18	hsa-miR-379*	87.59	2.99	−5.22
19	hsa-miR-99a	31.32	1161.16	5.21
20	hsa-miR-199a-5p	13.08	482.45	5.08
21	hsa-miR-214	273.73	9411.86	5.02
22	hsa-miR-382	525.03	19.72	−4.86
23	hsa-miR-195	29.20	774.93	4.78
24	hsa-miR-379	421.69	16.33	−4.70
25	hsa-miR-376a	154.50	6.80	−4.66
26	hsa-miR-31	19.84	453.26	4.64
27	hsa-miR-10a	166.79	7.61	−4.54
28	hsa-miR-495	432.48	19.53	−4.51
29	hsa-miR-125b	817.38	17,867.52	4.44
30	hsa-miR-10b	239.12	12.19	−4.34
31	hsa-miR-376c	152.76	8.74	−4.26
32	hsa-miR-127-3p	264.57	14.57	−4.21
33	hsa-miR-485-3p	201.48	11.46	−4.15
34	hsa-miR-134	253.52	16.61	−3.93
35	hsa-miR-654-3p	135.44	9.21	−3.89
36	hsa-miR-494	340.42	23.21	−3.88
37	hsa-miR-299-5p	63.59	4.52	−3.81
38	hsa-miR-346	142.45	11.14	−3.67
39	hsa-miR-483-5p	732.65	54.49	−3.63
40	hsa-miR-92b*	8.74	83.51	3.35
41	hsa-miR-1224-5p	242.56	26.22	−3.33
42	hsa-miR-218	206.82	18.48	−3.24
43	hsa-miR-708	96.69	10.88	−3.12
44	hsa-miR-411*	133.62	16.40	−3.11
45	hsa-miR-301a	19.88	178.36	3.06
46	hsa-miR-329	80.60	9.30	−3.05
47	hsa-miR-26b	333.25	2757.24	3.02
48	hsa-miR-486-5p	20.88	168.02	2.98
49	hsa-miR-923	3989.25	569.35	−2.84
50	hsa-miR-28-5p	19.80	144.71	2.81
51	hsa-miR-149*	428.72	74.95	−2.75
52	hsa-miR-505*	57.46	362.36	2.69
53	hsa-miR-485-5p	76.11	12.85	−2.68
54	hsa-miR-936	112.94	19.39	−2.62
55	hsa-miR-29a	78.94	460.97	2.61
56	hsa-miR-409-3p	69.31	11.71	−2.58
57	hsa-miR-1469	349.41	58.58	−2.57
58	hsa-miR-24	879.82	4759.30	2.45
59	hsa-miR-765	92.39	19.76	−2.44
60	hsa-miR-197	99.74	495.21	2.39
61	hsa-miR-99b	489.09	2103.62	2.11
62	hsa-miR-125a-5p	2121.30	9360.49	2.10
63	hsa-miR-23a	3198.24	13,081.13	2.02
64	hsa-miR-638	2752.88	699.84	−1.98
65	hsa-miR-1275	2714.17	677.40	−1.97
66	hsa-miR-27a	515.20	2034.19	1.96
67	hsa-miR-21	7236.65	26,700.40	1.84
**68**	**hsa-miR-574-5p**	**297.39**	**91.84**	**−1.70**
69	hsa-miR-324-5p	40.85	131.84	1.69
70	hsa-miR-663	341.14	128.67	−1.53
71	hsa-miR-222	583.09	1544.61	1.39
72	hsa-miR-421	156.91	367.95	1.32
73	hsa-miR-221	344.44	801.06	1.26
74	hsa-miR-27b	1254.00	2937.56	1.21
75	hsa-miR-23b	5303.07	12,176.63	1.20
76	hsa-miR-361-5p	2821.27	1247.87	−1.18
77	hsa-miR-877	352.14	789.34	1.17
78	hsa-miR-183	1885.45	4147.32	1.16
79	hsa-miR-1246	18,178.79	8399.43	−1.11
80	hsa-miR-191	4246.30	1992.85	−1.09
81	hsa-miR-128	490.61	1071.81	1.09
82	hsa-miR-130b	301.35	609.02	1.08
83	hsa-miR-1180	124.32	260.40	1.07
84	hsa-miR-15a	321.22	667.43	1.03
85	hsa-miR-92a	27,966.93	14,178.41	−1.00
86	hsa-miR-132	270.45	533.41	0.98
87	hsa-miR-20b	4633.50	2432.38	−0.95
88	hsa-miR-92b	16,350.76	8266.02	−0.94
89	hsa-miR-374b	1210.23	617.94	−0.93
90	hsa-miR-25	9060.44	4780.17	−0.92
91	hsa-let-7b	21,882.23	11,552.59	−0.91
92	hsa-let-7e	25,328.08	13,470.29	−0.91
93	hsa-miR-1308	11,466.81	6247.69	−0.88
94	hsa-miR-425	475.65	272.28	−0.80
95	hsa-let-7i	8834.50	15,112.88	0.78
96	hsa-miR-106b	1731.98	2891.30	0.77
97	hsa-let-7c	33,806.02	20,255.98	−0.75
98	hsa-let-7d	31,363.77	18,654.38	−0.75
99	hsa-miR-20a	10,936.77	6714.28	−0.68
100	hsa-miR-196a	2828.72	1772.16	−0.66
101	hsa-let-7f	32,482.40	20,436.54	−0.66
102	hsa-miR-342-3p	932.23	1390.13	0.63
103	hsa-miR-106a	8445.28	5478.16	−0.61
104	hsa-miR-720	482.17	732.43	0.60
105	hsa-miR-17	8763.89	5901.69	−0.59
106	hsa-miR-181a	1184.53	1782.06	0.59
107	hsa-miR-30b	1281.88	871.32	−0.55
108	hsa-miR-19b	1272.50	872.70	−0.54
109	hsa-let-7a	38,956.19	26,905.20	−0.52
110	hsa-miR-9	30,484.05	21,325.89	−0.52
111	hsa-let-7 g	5184.47	7483.50	0.51
112	hsa-miR-182	5809.37	8192.36	0.50
113	hsa-miR-423-5p	2331.59	3046.24	0.41
114	hsa-miR-1280	3304.08	4351.07	0.39
115	hsa-miR-93	2208.94	2880.50	0.39
116	hsa-miR-107	1519.29	1981.72	0.39
117	hsa-miR-16	8048.11	10,336.45	0.34
118	hsa-miR-320c	5008.38	3991.64	−0.32
119	hsa-miR-320b	4280.06	3403.00	−0.29
120	hsa-miR-320a	4926.46	3890.68	−0.29
121	hsa-miR-9*	7778.13	6630.25	−0.25
122	hsa-miR-1826	16,122.78	13,561.89	−0.24

**Fig. 4:**
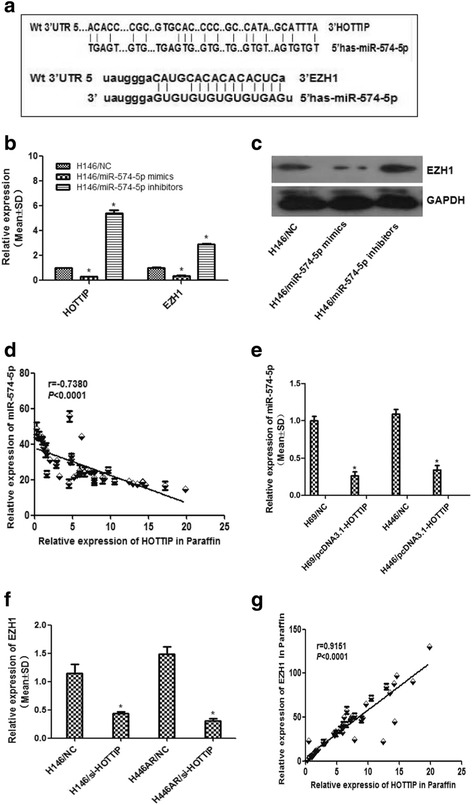
HOTTIP may enhance EZH1 expression by sponging miR-574-5p. **a** Putative binding site of miR-574-5p in HOTTIP and EZH1 3′-UTR and the site of target mutagenesis we indicated. **b** miR-574-5p may negatively regulated expression of HOTTIP and EZH1 at mRNA level in H146 cell. **c** miR-574-5p may negatively regulated expression of EZH1 at protein level in H146 cell. **d** & **g** Correlation analysis in SCLC tissues showed that HOTTIP expression is negatively associated with miR-574-5p but positively associated with EZH1 expression. **e** & **f** HOTTIP may positively regulated EZH1 expression but negatively regulated miR-574-5p. *, *P* < 0.05

Our previous studies have demonstrated that EZH1, EZH2 and other polycomb group genes had been proved to be involved in the development of SCLC even the occurrence of drug resistance. We also found that EZH1 contains putative regions that matches to the seed sequence of miR-574-5p but not match other miRNAs by further searching in RNA22-seq database (Fig. 4a). Hence, miR-574-5p was selected for further experiments in our study (Additional file 2: Fig. S2A). Our results verified that miR-574-5p negatively regulated the expression of HOTTIP and EZH1 by RT-qPCR and Western blot (Additional file 2: Fig. S2B-C, Fig. 4b-c), and co-expression relationship analysis of miR-574-5p and HOTTIP in clinical tissues showed a negative correlation (Fig. 4d). Moreover, HOTTIP overexpression led to an increase of miR-574-5p expression (Fig. 4e), while HOTTIP knockdown led to EZH1 decreasing significantly (Fig. 4f), and co-expression relationship analysis of EZH1 and HOTTIP in clinical tissues showed a positive correlation (Fig. 4g). These results above suggest HOTTIP may be involved in a novel regulatory network of miRNA-574-5p-HOTTIP-EZH1.

**Fig. 5:**
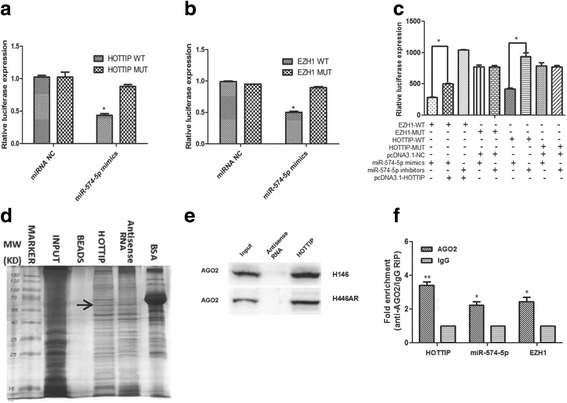
Further experiments confirmed the ceRNA role of HOTTIP. **a** & **b** & **c** Co-transfection dual luciferase reporter assay was used to demonstrate the ceRNA role of HOTTIP***.*** **d** & **e** RNA pull-down and SDS-page assay showed HOTTIP may interact with Ago2 protein. **f** RIP experiments showed that Ago2 had a function in the formation of HOTTIP and miR574-5p complex and in their regulation of EZH1. *, *P* < 0.05; **, *P* < 0.01

In order to clarify the role of HOTTIP in the novel regulatory complex, we carried out the co-transfection dual luciferase reporter assay and found that, luciferase activity for HOTTIP and EZH1 were reduced respectively compared with the control when miR-574-5p expressed (Fig. 5a-b), and the inhibited reporter plasmid luciferase activity for EZH1 was reversed in the presence of HOTTIP (Fig. 5c), which indicates that HOTTIP acts as an endogenous “sponge” by binding miR-574-5p, thus abolishing the miRNA-574-5p induced repressing activity on the EZH1 3′-UTR.

As we all know, miRNAs are present in the form of miRNA ribonucleoprotein complex within the cytoplasm, there the core component of RISC (RNA-induced silencing complex), Ago2 is involved. To detect whether HOTTIP is present in miRNPs, RNA pull-down, mass spectrometry and Western blot experiments were carried out and found that HOTTIP may interact with Ago2 protein directly (Fig. 5d-e, Table 3). Moreover, RIP experiments showed that Ago2 had a function in the formation of HOTTIP and miR574-5p complex and in their regulation of EZH1 (Fig. 5f), Which suggests HOTTIP, miR-574-5p and Ago2 combination formed the RNA silencing complex, and further clarify the direct interaction of miR-574-5p, HOTTIP and EZH1. These data above indicate that HOTTIP may modulate EZH1 expression as a ceRNA once more.

**Table 3:** Mass spectrometry results of the proteins pulled down by HOTTIP

Hits	Protein Name	Score	Mass	Matches	Sequences	emPAI
1	UBE2N_HUMAN	71	17,184	4 (2)	4 (2)	0.43
2	4F2_HUMAN	70	68,180	3 (1)	3 (1)	0.05
3	PGRC2_HUMAN	70	23,861	4 (2)	4 (2)	0.3
4	TCAL4_HUMAN	69	24,746	4 (2)	4 (2)	0.29
5	TM109_HUMAN	69	26,194	5 (3)	4 (3)	0.43
6	ACBP_HUMAN	68	10,038	3 (2)	3 (2)	0.82
7	CH60_HUMAN	68	61,187	7 (2)	7 (2)	0.11
8	RA1L2_HUMAN	67	34,375	7 (1)	5 (1)	0.1
9	RL29_HUMAN	67	17,798	3 (1)	3 (1)	0.19
10	PPIA_HUMAN	67	18,229	4 (2)	2 (2)	0.41
11	ZFP91_HUMAN	66	64,261	2 (1)	2 (1)	0.05
12	SC61B_HUMAN	66	10,025	1 (1)	1 (1)	0.35
13	LASP1_HUMAN	66	30,097	3 (2)	3 (2)	0.23
14	T22D1_HUMAN	65	109,649	4 (2)	4 (2)	0.06
15	WBP4_HUMAN	65	42,652	9 (1)	5 (1)	0.08
16	ECE1_HUMAN	65	87,906	5 (2)	5 (2)	0.08
17	ROA2_HUMAN	64	37,464	6 (2)	6 (2)	0.18
18	RS28_HUMAN	64	7893	1 (1)	1 (1)	0.45
19	BL1S2_HUMAN	64	16,008	3 (1)	3 (1)	0.21
20	MAP2_HUMAN	63	199,860	13 (1)	10 (1)	0.02
21	PP1RB_HUMAN	63	14,229	1 (1)	1 (1)	0.24
22	UB2V2_HUMAN	63	16,409	4 (2)	4 (2)	0.45
23	NFU1_HUMAN	63	28,615	3 (2)	2 (2)	0.25
24	TPBG_HUMAN	63	46,573	2 (2)	2 (2)	0.15
25	CR025_HUMAN	62	43,526	4 (1)	4 (1)	0.08
26	4EBP1_HUMAN	61	12,686	2 (2)	2 (2)	0.61
**27**	**AGO2_HUMAN**	**61**	**98,400**	**7 (1)**	**6 (1)**	**0.03**
28	I2BP2_HUMAN	60	61,728	2 (1)	2 (1)	0.05
29	RABP2_HUMAN	60	15,854	3 (2)	3 (2)	0.47
30	C1QBP_HUMAN	59	31,742	1 (1)	1 (1)	0.1
31	YLPM1_HUMAN	59	220,077	12 (2)	11 (2)	0.03
32	PSB9_HUMAN	58	23,364	2 (1)	2 (1)	0.14
33	BASI_HUMAN	58	42,573	2 (2)	2 (2)	0.16
34	COX5A_HUMAN	58	16,923	2 (1)	2 (1)	0.2
35	MAVS_HUMAN	58	57,063	1 (1)	1 (1)	0.06
36	BET1L_HUMAN	57	12,437	3 (1)	3 (1)	0.28
37	ATIF1_HUMAN	57	12,241	9 (2)	6 (1)	0.28

Abbreviations: Score, protein score; Mass, molecular weight of matched protein; Matches, figures in brackets indicate the number of matched peptides (*P* < 0.05); Sequences, type of peptide segment sequences which matches the respective peptide (*P* < 0.05); emPAI, index of protein abundance

It is necessary to add that, to better understand the relationship between HOTTIP and miR-574-5p and EZH1, we have evaluated cellular localization of HOTTIP in SCLC cells (Additional file 3: Fig. S3A). RNA was extracted from both nuclear and cytoplasmic fractions of cells separately, then HOTTIP was detected predominantly in the nuclear fraction as U6 RNA did in SCLC cells. Considering the exclusive localization of HOTTIP in the nuclei and Ago2 generally interacts with RNAs exported to cytoplasm, we examined if expression of ‘nuclear’ HOTTIP and EZH1 could be inhibited by miRNA-574-5p via RT-qPCR using nuclear and cytoplasmic RNAs isolated from cells treated with miR-574-5p. As shown in Additional file 3: Fig. S3B-E, we found that miRNA-574-5p could directly silence the expression of HOTTIP and EZH1 in either the nucleus or cytoplasm.

### HOTTIP may be involved in SCLC biology partly by positively regulating EZH1

**Fig. 6:**
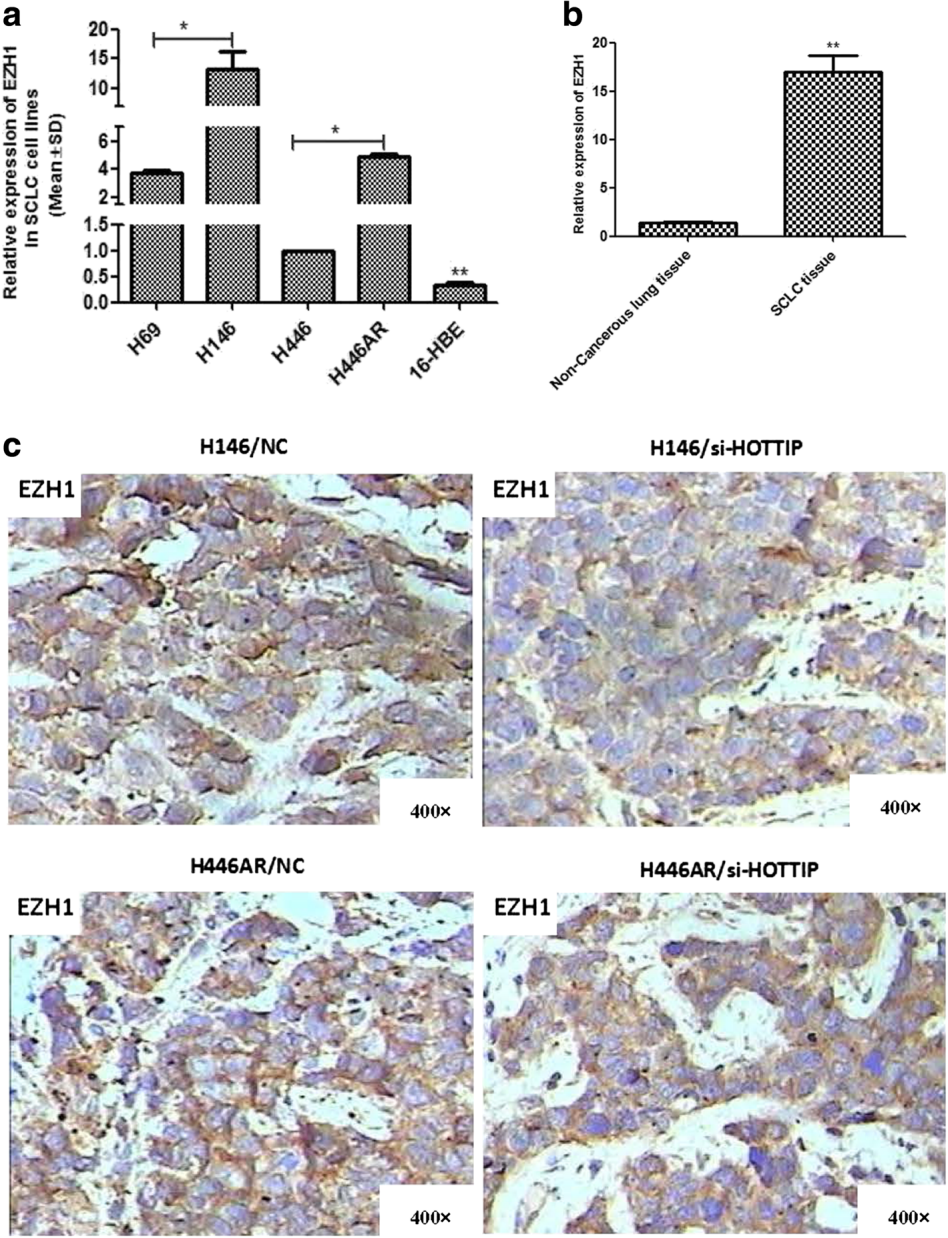
HOTTIP may be involved in SCLC progression partly by regulating EZH1. **a** Differential expression of EZH1 in SCLC cell lines and 16-HBE cell line by RT-qPCR. **b** Differential expression of EZH1 in SCLC tissues and non-tumoral lung tissues by RT-qPCR. **c** HOTTIP may positively regulate expression of EZH1 protein in xerograft tumor tissues by immunohistochemistry technique. *, *P* < 0.05

Further investigation showed a high expression of EZH1 in SCLC cell lines and clinical samples (Fig. 6a-b). Moreover, using immunohistochemistry technique, we confirmed the positively regulatory relationship between HOTTIP and EZH1 protein in the mice xenografts in vivo (Fig. 6c). Therefore, HOTTIP may be involved in SCLC pathogenesis by regulating EZH1.

### miR-574-5p may mediate the effect of HOTTIP on SCLC biology

**Fig. 7:**
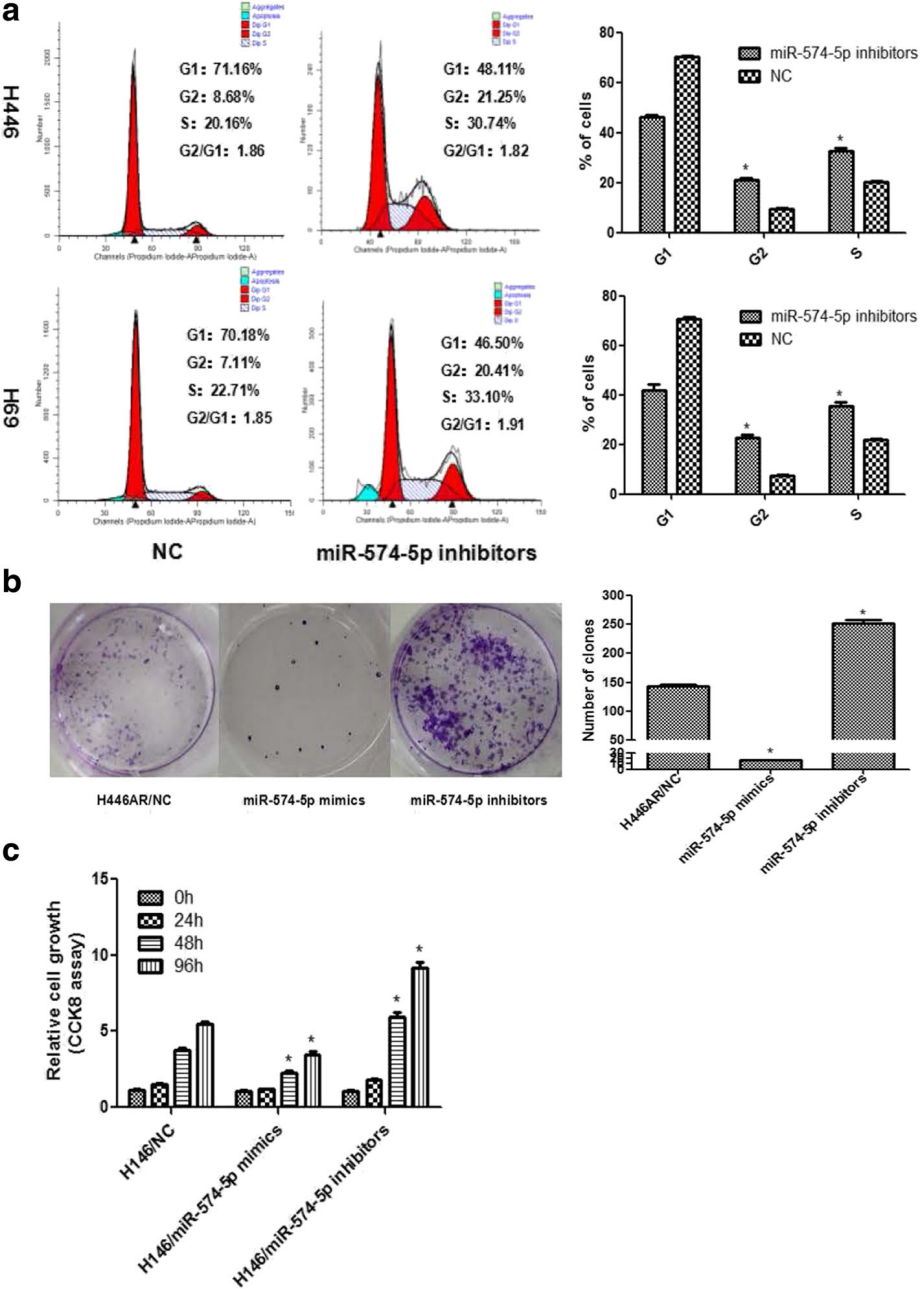
miR-574-5p may be involved in SCLC progression. **a** Flow-cytometric analysis was used for cell cycle detection after miR-574-5p suppression in H446 and H69 cells. **b** Plate clone formation assay showed miR-574-5p may inhibit SCLC cell proliferation. **c** CCK8 assay showed miR-574-5p may inhibit SCLC cell proliferation. *, *P* < 0.05

In the above section, we have proved the negative relevance between miR-574-5p and HOTTIP, but whether miR-574-5p is involved in SCLC biology is still unclear. By flow cytometry, cell-cycle profiles following miR-574-5p knockdown showed that suppression of miR-574-5p led to an increase in the number of cells in the S-phase and the G2-phase (Fig. 7a), which indicated that miR-574-5p suppression led to an opposite effect on SCLC cell cycle with HOTTIP knockdown. Plate colony formation experiments showed that miR-574-5p knockdown may promote SCLC cell growth while miR-574-5p overexpression may inhibit that (Fig. 7b). Using CCK8 assay, we found that miR-574-5p knockdown may promote SCLC cell growth while miR-574-5p overexpression may inhibit that (Fig. 7c).

Therefore, HOTTIP, miR-574-5p, EZH1 were all involved in the pathogenesis of SCLC according to the results above. As a vital role, HOTTIP may be involved in SCLC through the regulatory network “HOTTIP/miR-574-5p/EZH1”.

## Discussion

Recent studies have shown that dysregulated expression of lncRNAs in solid cancers reflects disease progression and may independently predict disease outcome [14, 15]. HOTTIP, which lies at the 5′ tip of the HOXA locus and drives H3K27me3 and gene transcription by binding with WDR/MLL complex [8], has been identified as a critical factor with tumor progression and drug resistance in pancreatic cancer and lung cancer without SCLC [16]. In addition, HOTTIP has been identified as a negative prognostic factor in hepatocellular carcinoma patients [17]. In the present study, we demonstrate that HOTTIP is associated with SCLC tumor progression and disease outcome.

This present study provides the first insight into the effect of HOTTIP on SCLC cell behavior. Our results demonstrate a pivotal role of HOTTIP in SCLC pathogenesis and prognosis by applying gain- and loss-of-function experiments in vitro *and* in vivo. Firstly, by detecting the expression of HOTTIP in 50 cases of human SCLC biopsy tissues, we found that HOTTIP was up-regulated in SCLC tissues in comparison with non-tumoral lung tissues. Further investigation in SCLC tissues showed that HOTTIP up-regulation was correlated with advanced disease stage and shorter median survival time. Therefore, our data suggests that HOTTIP is a novel independent prognostic factor for SCLC patients. Secondly, to explore the role of HOTTIP in SCLC biology, we carried out in vitro and in vivo experiments and found that HOTTIP overexpression promoted SCLC cell proliferation and colony formation while HOTTIP knockdown resulted in cell cycle arrest in G2 and S-phase, inhibited cell viability, colony formation in vitro and xenograft tumor growth in vivo. Hence, those findings above suggest HOTTIP may play a direct role in the modulation of multiple oncogenic properties and SCLC progression. Thirdly, further mechanistic investigations showed that HOTTIP might function as a role of ceRNA by binding miR-574-5p and abrogating their tumor suppressive function in this setting. Meanwhile, HOTTIP was involved in SCLC pathogenesis by up-regulating the expression of miRNA-574-5p’s target gene, EZH1, through competitively “sponging” this miRNA. Consequently, using a series of in vitro and in vivo experiments, our study suggested that HOTTIP is involved in SCLC tumorigenesis through the ceRNA network “HOTTIP/miR-574-5p/EZH1”.

In addition, we found that the HOTTIP knockdown could inhibit the expression of EZH1 protein in the mouse xenograft tissues except for its antagonistic effect on tumor growth, which also confirmed that the mechanism of HOTTIP involved in SCLC development possibly through positively regulating EZH1. Combining the above discussion, regulation of EZH1 expression by HOTTIP may be completed through the ceRNA mechanism, while comprehensive regulatory mechanism is still needed for further study.

In this study, dual luciferase reporter assay was used to confirm the existence of specific crosstalk between HOTTIP and EZH1 mRNA through competition for combining with miR-574-5p respectively. Furthermore, we used RNA pull-down, mass spectrometry and RIP assays to support the ceRNA mechanism of miR-574-5p and its target genes through modulating RISC. In addition, RT-qPCR assay revealed that EZH1 was mainly up-regulated in advanced stage SCLC tissues and associated with high HOTTIP expression. Altogether, the positive correlation between HOTTIP and EZH1 expression, and their relevance to miR-574-5p expression confirm our hypothesis that ceRNA may sequester miRNA, thereby protecting their target mRNAs from repression.

Although Ago2 commonly locates in cytoplasm, and mature miRNAs can be transported from cytoplasm to nucleus by importin 8 [18, 19]. Hence, there is essential machinery for RISC working in nucleus, which explains why HOTTIP mainly exists in the nuclei but could physically interact with Ago2. Similar regulation mechanisms of miRNAs were also observed in other nucleus lncRNAs [20–22].

Future work will validate HOTTIP as a predictive bio-marker for SCLC chemo-resistance, invasion and metastasis. A deeper characterization of the function and downstream signaling pathways influenced by HOTTIP deregulation is being investigated in our team, which may provide novel insights into the mechanisms of SCLC pathogenesis and possibly leading to the development of new therapeutic agents.

## Conclusions

Taken together, our study showed that HOTTIP was highly expressed in SCLC tissues, which was closely associated with clinical stage and overall survival in SCLC patients. Furthermore, the effects of HOTTIP on SCLC cell proliferation and cell cycle regulation indicated that HOTTIP could promote SCLC tumorigenesis. We also demonstrated that HOTTIP was involved in SCLC pathogenesis through ceRNA network “HOTTIP/miR-574-5p/EZH1”, then led to the occurrence and progression of SCLC. This study may provide a strategy and lead to the development of lncRNAs directed diagnostics and therapeutics against SCLC.

## Methods

### Human tissue specimens and cell culture

A total of 50 formalin-fixed, paraffin-embedded (FFPE) tissues were obtained from patients who had underwent bronchofiberscopy or biopsy for SCLC diagnosis during January 2008 to January 2015 and receiving care and follow-up at Shunde Hospital, Southern Medical University. The non-tumoral lung tissues in our study are all from the lung benign diseases including bronchiectasis and pulmonary bulla by thoracoscopic lobectomy. Informed consent was obtained from all patients and the study was approved by the Hospital’s Protection of Human Subjects Committee.

H146, H446, H69, H69AR and 16-HBE cell lines were got from the American Type Culture Collection (ATCC, USA), cultured in RPMI 1640 medium containing 10% or 20% fetal bovine serum in a humidified incubator at 37 °C with 5% CO_2_ respectively. The H446AR cell line was obtained by culturing H446 cell line in gradually increasing doses of adriamycin (ADM) up to 0.8 μM after a total of 14 months in our laboratory according to literature report [11]. The drug-resistant cells were maintained in drug-free medium for at least 2 weeks before any experiment. However, these drug resistant cell lines were also used for SCLC pathogenesis studies here.

According to the expression of HOTTIP in five cell lines above (Fig. 1c), we used H146/H446AR cell lines among which HOTTIP highly expressed to carry out loss-of-function experiments, while H446/H69 cell lines were used for gain-of-function experiments.

### Microarray analysis

For lncRNA microarrays (Arraystar company, USA) and miRNA microarrays (LCsciences), differentially expressed genes were ordered by *P*-value with a raw expression level over 400 folds. The detailed experimental procedures were performed as previously described [23].

### Overexpression and RNA interfere

The overexpression plasmid pcDNA3.1-HOTTIP was given as a present from Pro.Kevin Wang (Standford University School of Medicine). The pcDNA3.1-NC plasmid, siRNAs/shRNAs and miRNA mimics/inhibitors/antagomirs were purchased from GenePharma. The effective interference sequences were all selected by RT-qPCR for the best gene silencing effect and then used for subsequent experiments. For stable transfection, positive transfectants were selected with 400 μg/ml G418 (Calbiochem), while HOTTIP siRNAs were pakaged by lentivirus. The related siRNAs, shRNAs or miRNA mimics/inhibitors/antagomirs sequences were listed in Additional file 4.

### Reverse transcription quantitative PCR (RT-qPCR)

RT-qPCR was used to detect expression levels of HOTTIP and other genes in SCLC cancer tissues and cells according to the manufacturer’s instructions (TAKARA). GAPDH was used as the control. Related primers are listed in Additional file 4.

### Western blot and immunohistochemistry staining (IHC) analysis

Western blot and immunohistochemistry staining (IHC) analysis were performed according to standard protocols as described previously [8, 13]. All antibodies information is listed in Supplemental material.

### RNA pull-down assay

Briefly, biotin-labeled RNAs were transcribed in vitro with the Biotin RNA Labeling Mix (Roche) and T7 RNA polymerase (Roche), treated with RNase-free DNase I (Roche), and purified with the RNeasy Mini Kit (Qiagen). The procedure was carried out according to the manufactures’s instructions and standard protocols as described previously [24].

### Mass spectrometry

HOTTIP and antisense strand protein bands acquired by RNA pull-down assay were excised and examined by mass spectrometry to detect the related protein combined directly with HOTTIP. The procedure was carried out according to standard protocols described previously [19].

### RNA immunoprecipitation (RIP)

RIP assay is used to check whether miRNAs regulating their target genes trough RISC. The MS2bs-MS2bp-based RIP assay was carried out according to previous reports [25].

### In vitro proliferation assay

Cell counting kit 8 (CCK8) experiment for in vitro proliferation assay were performed using the CCK8 Kit (Dojindo) according to the manufacturer’s instructions as described previously [26].

For soft agar colony formation (for suspension-cultured H69 cell) and plate colony formation experiments, they were carried out according to standard protocols described previously [27].

### In vivo SCLC xenograft model in nude mice

SCLC xenograft tumor formation experiment in nude mice was carried out according to the institutional guidelines of Guangdong Province and being approved by the Use Committee for Animal Care. Twenty-four BALB/c nude mice (male, 5–6 weeks old, 18.0 ± 0.5 g) were got from the Guangdong Medical Animal Center, this experiment was carried out at the Animal Experimental Department of Sun Yat-sen University North District. They were randomly divided into the following groups (*n* = 6 mice per group): (a) H146/NC; (b) H146/si-HOTTIP; (c) H446AR/NC; (d) H446AR/si-HOTTIP. H146 and H446AR cells with stable HOTTIP knockdown by lentivirus infection were injected into the flanks of nude mice at a concentration of 1 × 10^7^ cells per 0.2 ml. The tumors were measured every 3 or 4 days, and tumor volume was calculated using the following formula: volume = (L × W^2^)/2, among which L and W are the longest and shortest diameters, respectively. The mice were sacrificed when the average L of any group reached approximately 1 cm.

### Flow cytometric analysis for cell cycle

Cell cycle assay were performed after cells being fixed in 70% ethanol overnight at 4 °C, and then were stained with propidium iodide. The detailed procedure was carried out according to standard protocols described previously [22].

### Luciferase reporter assay

PSICHECK2.0 plasmid encoding a luciferase reporter gene was purchased from Promega. Recombinant plasmid of PSICHECK2.0-H-HOTTIP-3′-UTR, PSICHECK2.0-H-EZH1–3′-UTR (wild type) or corresponding mutant type were constructed in GenePharma. SCLC cells (2 × 10^5^ cells/well) were spread in a 12-well plate and co-transfected with 40 nM of either hsa-miR-574-5p or miRNA negative control of either recombinant plasmids or corresponding mutants, and 1 ng of PSICHECK2.0 (Promega) by using Lipofectamine™ 2000. The PSICHECK2.0 vector was used as an internal control to correct the differences in both transfection and harvest efficiency. Cells were collected 48 h after transfection and analyzed using the dual luciferase reporter assay system (Beyotime Biotechnology).

### Statistical analysis

All experiments were run in triplicate. Data were represented as Mean ± Standard Deviation (SD). All statistical analyses were carried out using SPSS 13.0 Statistics Software. *P* < 0.05 was considered significant.

## Additional files

Additional file 1: HOTTIP and miR-574-5p were screened out by microarray and RT-qPCR methods. (JPEG 275 kb)

Additional file 2: HOTTIP regulated the cell cycle of SCLC cells. (JPEG 353 kb)

Additional file 3: Cell location of HOTTIP and verification of their targeted regulatory relationships in cytoplasm and nucleus. (JPEG 107 kb)

Additional file 4: Legends of supplementary figures, primers, RNAi sequences, miRNA and antibodies information. (DOC 633 kb)

## Abbreviations

ceRNA: Competing endogenous RNA
FFPE: Formalin-fixed, paraffin-embedded
H3K27me3: Histone H3 lysine 27 trimethylation
HOTTIP: HOXA transcript at the distal tip
LncRNA: Long non-coding RNA
PRC2: Polycomb Repressive Complex 2
RIP: RNA binding protein immunoprecipitation
RT-qPCR: Reverse transcription quantitative PCR
SCLC: Small cell lung cancer
