# Supplementary figures and images for: A long non-coding RNA HOTTIP expression is associated with disease progression and predicts outcome in small cell lung cancer patients
Source: Mol Cancer. 2017 Oct 17;16:162. doi: 10.1186/s12943-017-0729-1 (PMC5646126; doi:10.1186/s12943-017-0729-1)

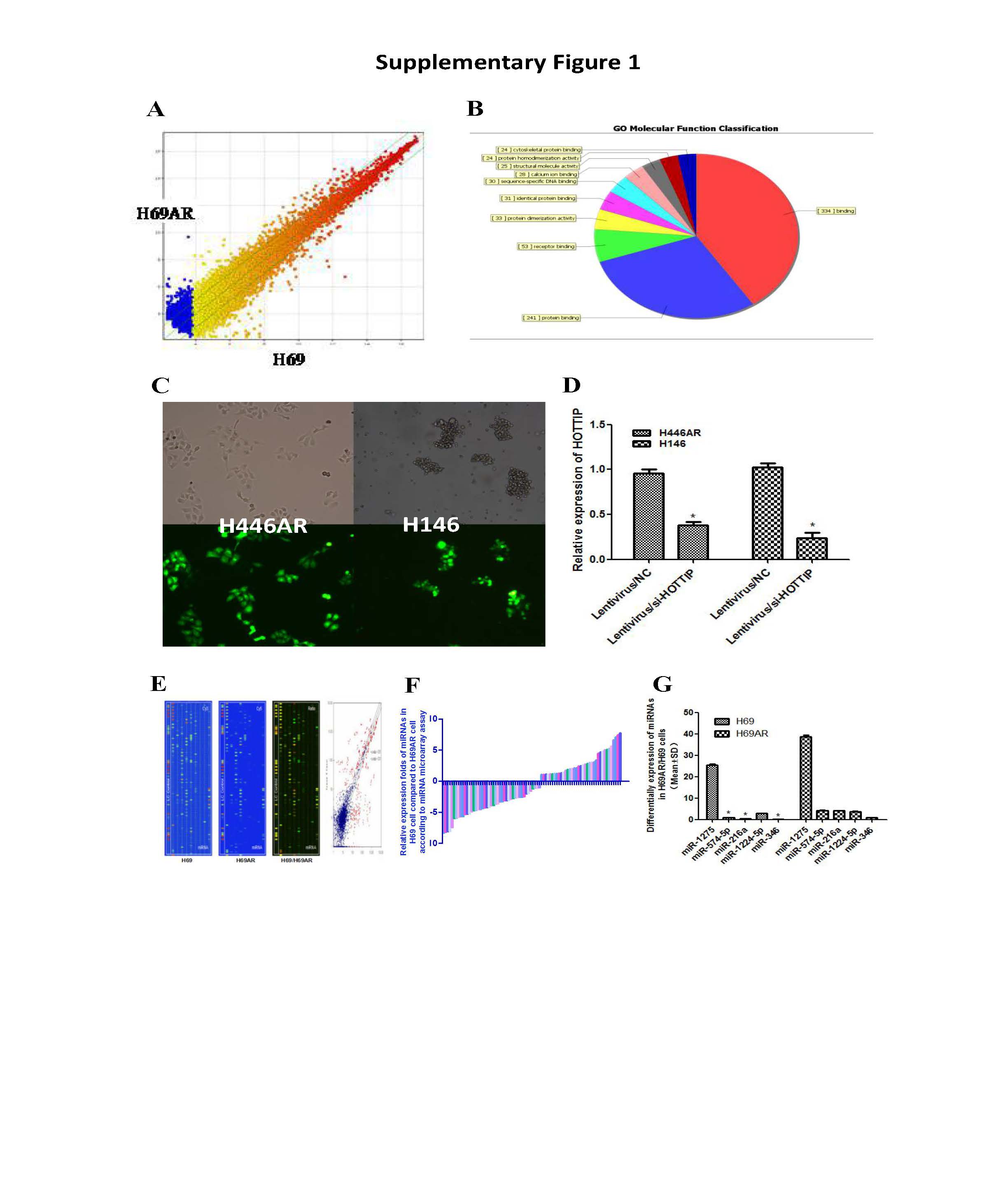

Supplement: Supplementary file 1 — HOTTIP and miR-574-5p were screened out by microarray and RT-qPCR methods. (JPEG 275 kb) [file 12943_2017_729_MOESM1_ESM.jpg]

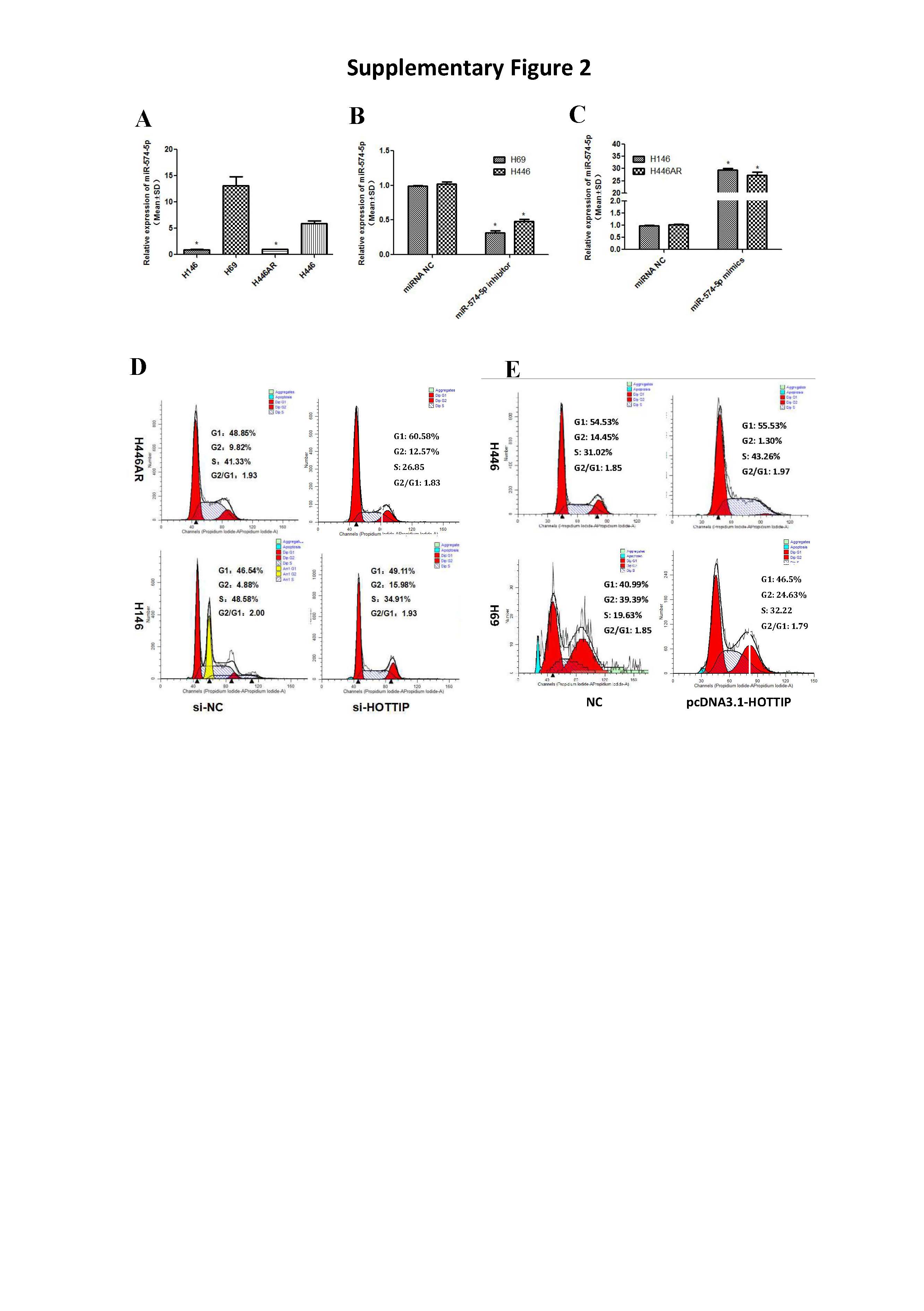

Supplement: Supplementary file 2 — HOTTIP regulated the cell cycle of SCLC cells. (JPEG 353 kb) [file 12943_2017_729_MOESM2_ESM.jpg]

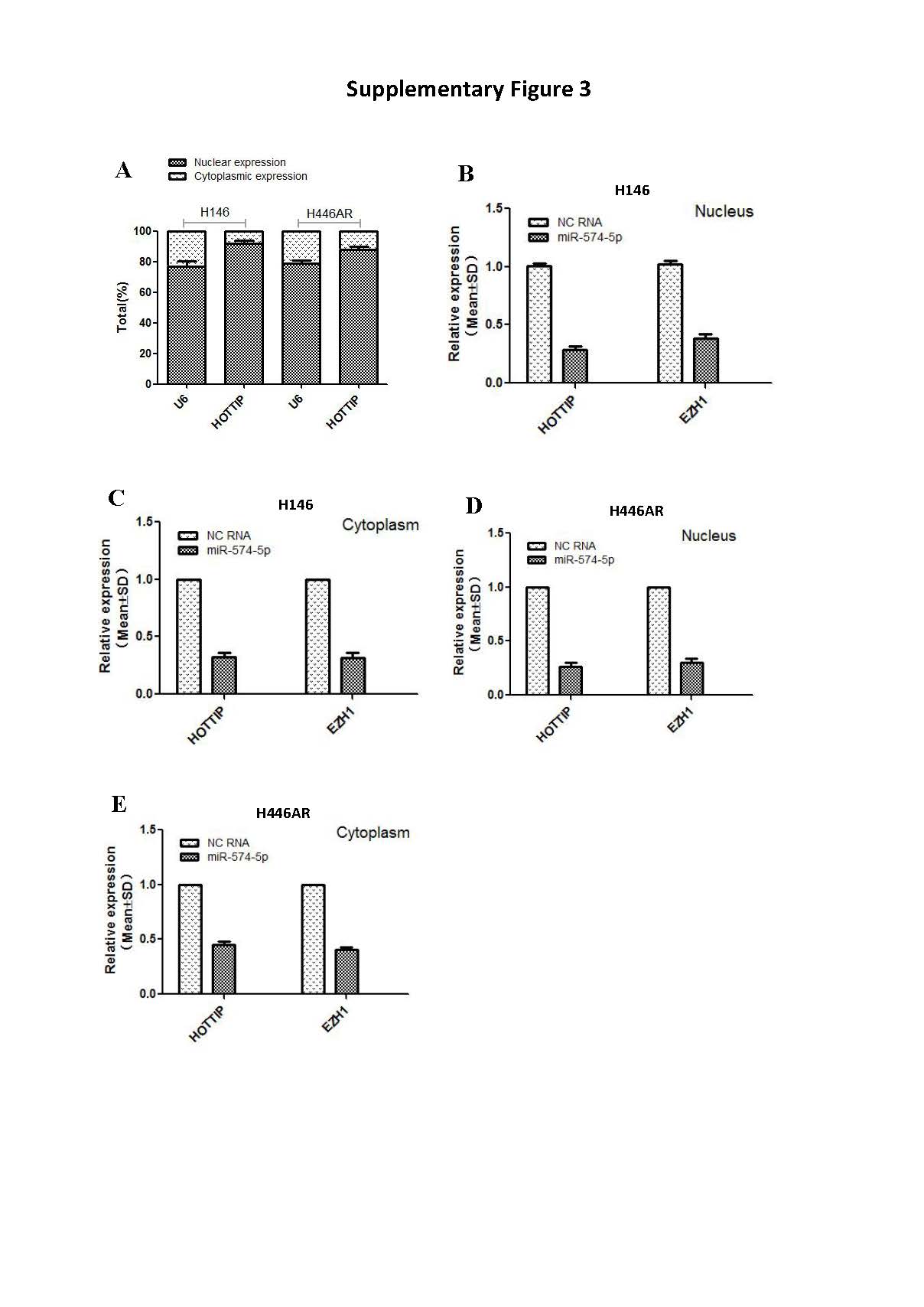

Supplement: Supplementary file 3 — Cell location of HOTTIP and verification of their targeted regulatory relationships in cytoplasm and nucleus. (JPEG 107 kb) [file 12943_2017_729_MOESM3_ESM.jpg]
