# Supplementary material for: A long non-coding RNA HOTTIP expression is associated with disease progression and predicts outcome in small cell lung cancer patients
Source: Mol Cancer. 2017 Oct 17;16:162. doi: 10.1186/s12943-017-0729-1 (PMC5646126; doi:10.1186/s12943-017-0729-1)
Supplement: Supplementary file 4 — Legends of supplementary figures, primers, RNAi sequences, miRNA and antibodies information. (DOC 633 kb) [file 12943_2017_729_MOESM4_ESM.doc]

**Supplementary Figure 1 legend:**

1. Analysis of differential expression of HOX genes in H69AR and H69 cell lines.
2. Biological function distribution of HOX genes by pie graph.
3. Green fluorescence of lentiviral vector transfected cells by electron fluorescence microscopy technique.
4. Changes of HOTTIP expression after transfection of lentiviral vector in H44AR and H146 cells.
5. MiRNA microarray results showed that 61 miRNAs may be related with SCLC progression.
6. Among them, 24 miRNAs were significantly higher than that in H69 cells, 37 miRNAs including miR-574-5p were significantly higher than that in H69AR cells.
7. Five miRNAs were screened out for their respective interaction with HOTTIP promoter binding sites by bioinformatics website RNA22-seq analysis.

**Supplementary Figure 2 legend:**

1. The expression of miR-574-5p in 4 SCLC cell lines.
2. The transfected effects of miR-574-5p inhibitors in H69/H446 cell lines.
3. The transfected effects of miR-574-5p mimics in H446AR/H146 cell lines.
4. Flow-cytometric analysis was used for cell cycle detection after HOTTIP knockdown in H146 and H446AR cells.
5. Flow-cytometric analysis was used for cell cycle detection after HOTTIP over-expression in H69 and H446 cells.

**Supplementary Figure 3 legend:**

(A) Cellular localization of lncRNA HOTTIP in SCLC cells.

(B) & (C) MiRNA-574-5p could directly silence the expression of HOTTIP and EZH1 in either nucleus or cytoplasm in H146 cell.

(D) & (E) MiRNA-574-5p could directly silence the expression of HOTTIP and EZH1 in either nucleus or cytoplasm in H446AR cell.

**Supplementary Table legends**

Table. 1. Clinicopathological data of the SCLC studied cohort.

Table. 2. Differentially expressed miRNAs in H69/H69AR cells.

Table. 3. Mass spectrometry results of the proteins pulled down by HOTTIP.

**Primers:**

HOTTIP Foward Primer(5’-3’): CCTAAAGCCACGCTTCTTTG

HOTTIP Reverse Primer(5’-3’): TGCAGGCTGGAGATCCTACT

EZH1 primers: Forward 5’-ATGCGACTTCGACAACTTAAACG-3’

Reverse 5’-GGCTTCATTGACTGAACAGGTT-3’

GAPDH primers: Forward 5′-GGGCTGCTTTTAACTCTG-3′

Reverse 5′-TGGCAGGTTTTTCTAGACGG-3′

**siRNA and shRNA sequences：**

si-h-HOTTIP-1:

(positive-sense, 5'-3'): GCUGCUUUAGAGCCACAUA dTdT

(negative-sense, 3'-5'): dTdT CGACGAAAUCUCGGUGUAU

si-h-HOTTIP-2:

(positive-sense, 5'-3'): CCAGCUGCGAAUUCUUAAU dTdT

(negative-sense, 3’-5’):dTdT GGUCGACGCUUAAGAAUUA

si-h-HOTTIP-3:

(positive-sense, 5'-3'):CCUUGAUAUGCACGCAUAU dTdT

(negative-sense, 3’-5’): dTdT GGAACUAUACGUGCGUAUA

HOTTIP lentivirus vector (si-h-HOTTIP-1 was packaged by LV3 lentivirus vector):

Vector type: LV3（H1/GFP&Puro） sequence: 5’ GCUGCUUUAGAGCCACAUA dTdT 3’

EZH1 shRNA sequences：

EZH1-homo-215: (sense, 5'-3'): 5′- GCGACUUCGACAACUUAAATT -′3

(anti-sense, 5'-3'): 5’- UUUAAGUUGUCGAAGUCGCTT - 3’

EZH1-homo-905: (sense, 5'-3'): 5′- GGAGAGGUAUCGAGAACUATT -′3

(anti-sense, 5'-3'): 5’- UAGUUCUCGAUACCUCUCCTT - 3’

EZH1-homo-2040: (sense, 5'-3'): 5′- GUGCAGAAGAACGAAUUCATT -′3

(anti-sense, 5'-3'): 5’- UGAAUUCGUUCUUCUGCACTT - 3’

**Sequencing results of pcDNA3.1-HOTTIP expression:**


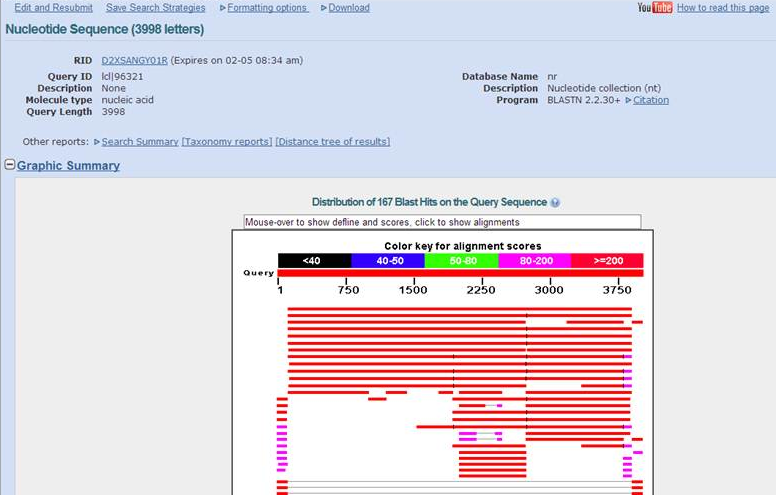


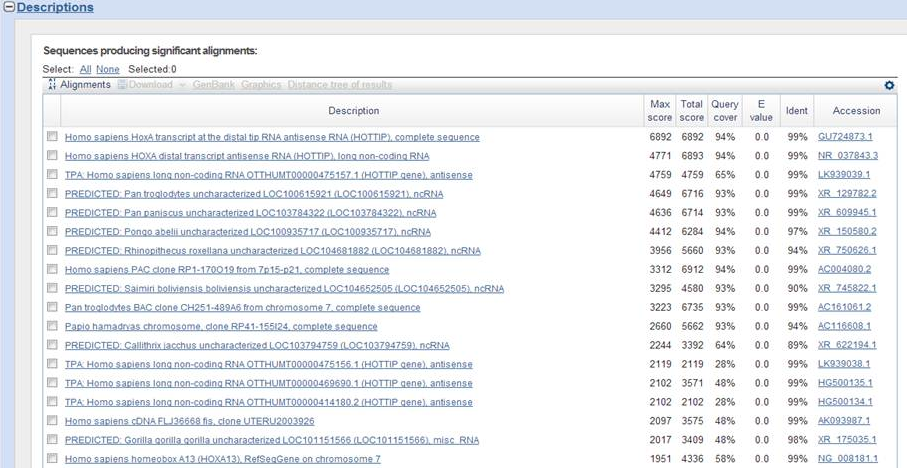


**miRNA mimics and inhibitor sequences：**

hsa-miR-574-5p mimics sequence:

UGAGUGUGUGUGUGUGAGUGUGU ACACUCACACACACACACUCAUU

hsa-miR-574-5p inihibitor sequence: ACACACUCACACACACACACUCA

hsa-miR-574-5p anragomir sequence: ACACACUCACACACACACACUCA

**Antibodies information:**

Rabbit anti-human EZH1 monoclonal antibody (Abcam, ab137693)
